# Retraction: Small Intestinal Tumors: A Rare Case of Tubulovillous Adenoma in Duodenum 

**DOI:** 10.7759/cureus.r15

**Published:** 2019-08-06

**Authors:** Mustafa N Malik, Zunairah M Shah, Abdul Rafae, Tayyab Mahmood, Hafiz M Fazeel

**Affiliations:** 1 Internal Medicine, District Headquarter Hospital, Rawalpindi, PAK; 2 Hematology and Oncology, District Headquarter Hospital, Rawalpindi, Pakistan, Rawalpindi, PAK; 3 Internal Medicine, Federal Government Services Hospital, Rawalpindi, PAK; 4 Hematology and Oncology, The University of Arizona, Tucson, USA

This article has been retracted at the request of the authors. A preliminary version of the article was previously published in an offline journal (and thus undetected by plagiarism software) and an Open Access image was also not properly credited by the authors nor disclosed to Cureus editorial staff. Proper image credit should have stated: https://en.m.wikipedia.org/wiki/Tubulovillous_adenoma.

